# Can TElemedicine system replace doctor consultations to Achieve non-inferior blood pressure in patients with Controlled Hypertension (TEACH)? Study protocol for a randomised controlled trial

**DOI:** 10.1186/s13063-025-09350-3

**Published:** 2025-12-08

**Authors:** Sze Nok Ng, Benjamin Hon-Kei Yip, Shuqi Wang, Maria Leung, Shirley Yue Kwan Choi, Shuk-yun Leung, Jessica Jinghao Han, Wendy Wing-Sze Tsui, Sum Yin Lai, Linda Chan, Anastasia S. Mihailidou, Richard J. McManus, Jimmy Sy, Eric Kam-Pui Lee

**Affiliations:** 1 The Jockey Club School of Public Health and Primary Care, The Chinese University of Hong Kong, Hong Kong SAR, China; 2 Department of Family Medicine, New Territory East Cluster, Hospital Authority, Hong Kong SAR, China; 3 Family Medicine & Primary Healthcare, Queen Mary Hospital, Hospital Authority, Hong Kong SAR, China; 4 Department of Family Medicine and Primary Care, Li Ka Shing Faculty of Medicine, The University of Hong Kong, Hong Kong SAR, China; 5 The Bau Institute of Medical and Health Sciences Education, Li Ka Shing Faculty of Medicine, The University of Hong Kong, Hong Kong SAR, China; 6 Department of Family Medicine and Primary Care, The University of Hong Kong-Shenzhen Hospital, Shenzhen, Guangdong China; 7 Department of Cardiology and Kolling Institute, Royal North Shore Hospital, Sydney, NSW Australia; 8 Brighton and Sussex Medical School, University of Brighton and University of Sussex, Brighton, UK

**Keywords:** Telemedicine, Hypertension, Chinese, Mobile app, Randomised controlled trial, TEACH, Blood pressure

## Abstract

**Background:**

Hypertension is the most prevalent chronic condition and is the leading cause of cardiovascular diseases, imposing enormous burdens on the healthcare system. Although telemedicine may provide improved blood pressure (BP) monitoring and control, it remains unclear whether it could replace face-to-face consultations for patients with optimal BP control. Thus, the primary objective of this study is to investigate whether participants assigned to the telemedicine group show non-inferior BP control compared to the usual care group at 12 months.

**Methods:**

This randomised controlled trial (RCT) will involve 364 patients receiving anti-HT medications who have well-controlled BP on out-of-office BP measurements, including HBPM or ambulatory blood pressure measurements (ABPM). Participants will be randomised to either the telemedicine (HealthCap) group or usual care (control) group (1:1). Patients in the intervention group will measure and transmit their 7-day home BP measurements (HBPM) to the physician’s office. The medications will be refilled without consultation when optimal control (<135/85 mmHg for patients without comorbidities and <130/80 mmHg for patients with comorbidities that increase cardiovascular risk) and safety questions are confirmed. Nevertheless, if any of the answers in the safety questions are positive or the HBPM mean is suboptimal, patients will have consultations as planned. Investigators will be blinded to the randomisation sequence and allocation. The primary outcome is the daytime ABPM systolic BP at 12 months. Secondary outcomes include HT treatment adherence, self-efficacy, number of visits to primary care clinics where they have clinical follow-up, health care utilisation other than general outpatient clinics (GOPCs) in both arms at baseline, 6 months, and 12 months. Acceptability will be assessed through interviews with the telemedicine study participants and the physician.

**Discussion and significance:**

This trial will examine whether patients in the telemedicine group would have non-inferior BP control compared to patients in the usual care group. It has the potential to change clinical practice and have important research implications because patients with optimal BP can monitor their BP through a telemedicine system and will have fewer frequent clinical visits. This will empower primary care and allow effective and safe allocation of scarce medical resources to patients in need. Moreover, it will save patients time because of the long wait to see doctors and collect medications in GOPCs. It is also encouraging the engagement of patients in their health because they will play a proactive role in managing their chronic illnesses.

**Trial registration:**

ClinicalTrials.gov NCT06524180. Registered on July 29, 2024. https://clinicaltrials.gov/study/NCT06524180?term=NCT06524180&rank=1#more-information.

**Supplementary Information:**

The online version contains supplementary material available at 10.1186/s13063-025-09350-3.

## Background

Hypertension (HT) is the most prevalent chronic condition (affecting approximately 30% of the world’s and Hong Kong’s [HK] adult population) and is the leading cause of cardiovascular diseases and death. Although approximately 50% of patients with HT in HK have optimal blood pressure (BP) control, these patients have doctors’ consultations every 16–18 weeks to monitor BP control and refill medications [1]. Owing to an ageing population and improved HT screening programme in HK, the number of HT patients requiring medical attention will increase and overload our primary care healthcare system. For instance, the recently established primary healthcare office will provide universal HT screening for all residents aged ≥45 years, while currently, approximately 50% of patients with HT are undiagnosed [2]. Therefore, a timely and novel strategy that can confirm good BP control and automatically refill medications in patients with good HT control is urgently needed to allocate healthcare resources better and empower HK primary care and patients.

Telemedicine is defined as the use of technology which allows the automatic exchange of medical information (i.e. BP readings) between patients and healthcare providers to manage diseases at a distance [3]. Since telemedicine allows confirmation of good home BP control without in-person consultations and drug refills, it has great potential to replace in-person doctor consultations and reallocates these resources to patients with more significant health needs [3]. In fact, telemedicine has improved patients’ BP in randomised controlled trials (RCTs) and can be cost-effective [4, 5]. A recent meta-analysis reported a 4 mmHg and 2 mmHg reduction in systolic BP (SBP) and diastolic BP (DBP), respectively, in patients receiving telemedicine. Telemedicine enhanced patients’ self-efficacy and treatment adherence by encouraging regular BP monitoring and self-management [6, 7].

However, the clinical role of telemedicine in patients with optimally controlled BP is unclear because existing RCTs included patients with elevated BP, and telemedicine typically represented more intensive treatments in these RCTs [3–7]. The ideal frequency of telemedicine monitoring in patients with optimal BP control remains unknown [3]. There is also uncertainty whether telemedicine can reduce or replace clinic visits, with limited relevant evidence [3]. Similarly, the cost and safety of telemedicine are under-reported in existing RCTs [3]. Importantly, the role and effectiveness of telemedicine are understudied in Chinese. With the latest meta-analysis on telemedicine and HT only including one Chinese RCT (*n* = 59), ranked as having a high risk of bias [6]. Understanding the factors to enhance the effectiveness and successful implementation of telemedicine systems, such as taking into consideration different cultures, patients’ age and education, and healthcare system organisations, is vital [8].

To examine the feasibility of using a telemedicine system to reduce doctor consultations, we conducted a 6-month pilot RCT (*n* = 49) in 2020 which randomised patients with optimal BP control to a telemedicine arm or a usual care arm [9]. The telemedicine system (called “HealthCap”) used in the pilot RCT was developed by our team. It can record home BP measurements (HBPM), provide automatic feedback on different BP levels, transfer BP data to the case physicians, and confirm optimal BP control on HBPM (details under methods) [9]. For patients randomised to the HealthCap intervention, drugs were prescribed in the clinic without doctor consultation, and the index physician’s consultation was deferred after optimal BP control was confirmed by HealthCap. Our pilot RCT found that (i) patients’ retention was high (98%), (ii) patients’ acceptability was high, which was further confirmed by patients’ interviews (interviewees reported that HealthCap was convenient, time-saving, cost-saving, and educational), (iii) no side effects or hospitalisation was detected in both groups, (iv) similar SBP/DBP control between the two groups at 6-month follow-up (systolic BP: 128.2 versus 126.9 mmHg [telemedicine versus usual care], *p* = 0.41), and (v) clinical visits were reduced in the telemedicine group (0.8 versus 2 consultations, *p* < 0.001) [9]. Our pilot RCT also confirmed the feasibility of using HealthCap in general outpatient clinic (GOPC) settings [9].

Despite the encouraging results from our pilot RCT, these results should be confirmed in an adequately powered RCT. Furthermore, a cost-minimisation analysis (i.e. cost saving from using HealthCap) should be performed if HealthCap is non-inferior to usual care. HealthCap can be cost-saving for healthcare systems (i.e. due to fewer consultations) and patients because it can reduce absence from work due to clinical visits and medication refills (which often take 3–4 h in GOPC). Moreover, telemedicine systems should also undergo formal validation prior to widespread implementation because they are considered medical devices, and medical societies recommend formal validation [3]. Finally, most existing telemedicine systems have not been validated, and a validated telemedicine system for HT is lacking in HK [10]. Validated HT apps/systems in Western countries are not available in Chinese, which is the only language most HK elderly can read and furthermore the health-related information may be irrelevant (e.g. not tailored to locally available clinics or medical resources).

## Aims and objectives

The aim of the study is to investigate if a telemedicine system can replace doctor consultations to achieve non-inferior blood pressure in patients with controlled hypertension. The primary objective is to evaluate whether patients assigned to the telemedicine (HealthCap) group demonstrate non-inferior BP control compared to patients in the usual care group at 12 months. Secondary objectives include (i) assessing patients’ adherence, self-efficacy, number of primary care clinic visits, and healthcare utilisation in both arms; (ii) assessing acceptability to physicians and patients in the telemedicine group; and (iii) conducting a cost-minimisation analysis if HealthCap is non-inferior.

## Methods

### Trial design and patient involvement

This protocol was written with reference to the Standard Protocol Items: Recommendations for Interventional Trials (SPIRIT) [11]. The SPIRIT checklist can be found in additional document 1. This is a parallel-arm RCT in which 364 patients with optimally controlled HT will be randomised in a 1:1 ratio to the HealthCap telemedicine (intervention) and usual care (control) groups and followed up for 12 months. For the primary outcome, we hypothesised that patients randomised to the telemedicine (HealthCap) group would have non-inferior BP control compared to patients allocated to the usual care group at 12 months. The RCT was pre-registered on clinicaltrials.gov (NCT06524180) and was approved by the Joint Chinese University of Hong Kong-New Territories East Cluster Clinical Research Ethics Committee (2023.525) and The University of Hong Kong/Hospital Authority Hong Kong West Institutional Review Board (UW25-086). The study complies with the Declaration of Helsinki.

Conducted during the peak of the COVID-19 outbreak in HK, the recruitment rate of the pilot trial was suboptimal for a main RCT (8 participants/month by 2 recruiting doctors) [9]. Thus, necessary modifications were made to ensure adequate recruitment in this trial.

### Trial setting

Patients will be actively recruited by doctors, nurses, research assistant(s), and posters in GOPCs at New Territories East cluster and Hong Kong West cluster in Hong Kong. The detailed list of participating recruitment sites can be found in additional document 2.

### Recruitment and participant timeline

Our recruitment methods include in-person visits to recruitment sites, written materials (such as posters and leaflets), and referrals. Healthcare providers will identify potential patients during their follow-ups on hypertension and introduce the programme. All hypertension patients will receive periodic assessments under the Risk Assessment and Management Programme for Hypertension (RAMP-HT), during which the research team will directly approach them to discuss the project. Patients who see the posters and express interest in the trial can also register using the QR code provided in the poster or contact the research assistants directly through the communication tool (i.e. WhatsApp). Any issues with the registration process will be promptly addressed by our research team.

Interested patients will be contacted by the research team and undergo an eligibility screening. To standardise our recruitment approach, we conduct staffing training and hold meetings with healthcare providers in the GOPCs involved before patient enrolment. We also continuously engage doctors in each recruiting GOPC to encourage referrals of patients with well-controlled hypertension. Once patients are deemed eligible, they will be asked to sign informed consent online. Furthermore, the research team has developed strategies to ensure adequate enrolment and achieve the target sample size, including monitoring recruitment rates monthly and addressing challenges through regular meetings with healthcare providers. Patients will then be invited to the data collection session, during which all baseline measures will be assessed, patients will be randomly assigned either to the experimental group or the control group (T_0_, baseline). Afterwards, patients will be assessed at baseline (T_0_), 24 weeks (T1), and 1 year (T2) (Table 1).

**Table 1:**
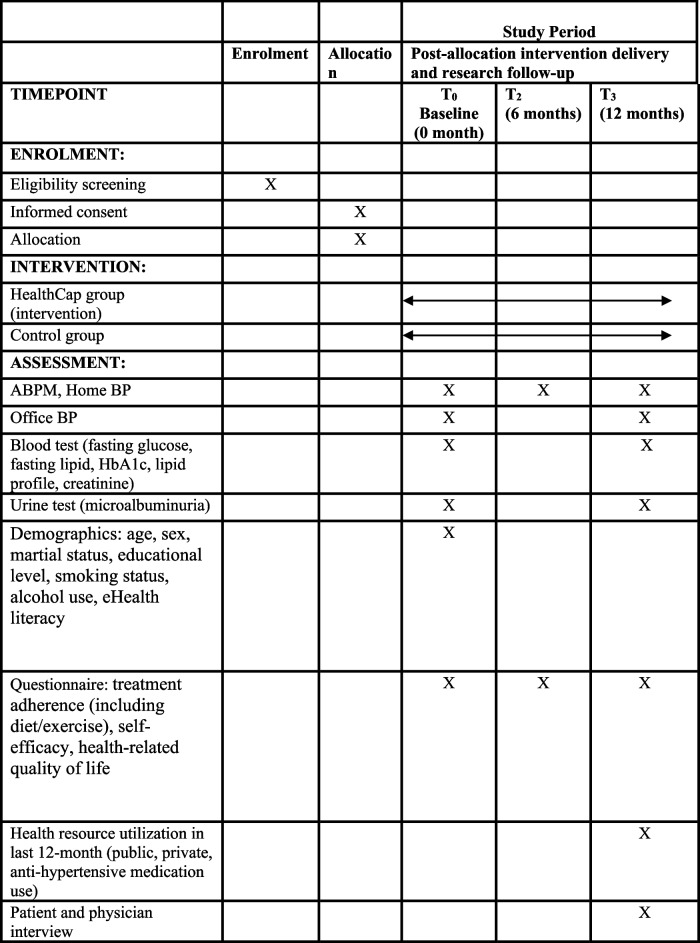
Schedule of enrolment, interventions, and assessments

*ABPM* ambulatory blood pressure monitoring, *BP* blood pressure, *HbA1c* glycosylated haemoglobin, *ACR* albumin-to-creatinine ratio

### Eligibility criteria

Patients who meet *all* these criteria will be included: (i) having a diagnosis of essential HT; (ii) on anti-HT medications; (iii) well-controlled HT on out-of-office BP measurements, including HBPM or ambulatory blood pressure measurements (ABPM) (measurement algorithm and details under methods) [12]. ABPM or HBPM are preferred to office BP due to their superior reproducibility and predictivity to cardiovascular outcomes [13, 14]. Furthermore, office BP misclassifies 30–40% of patients as having suboptimal BP control due to white-coat effect [15]. From our pilot study, some patients with optimal BP are reluctant to undergo ABPM before recruitment into the RCT, and HBPM is more acceptable to these patients and is therefore included. According to local and international guidelines, optimal out-of-office daytime BP should be <135/85 mmHg for patients without comorbidities and <130/85 mmHg for patients with comorbidities that increase cardiovascular risk (i.e. stroke, ischaemic heart diseases, heart failure, diabetes mellitus (DM), and chronic kidney diseases) respectively [12, 16]; (iv) can read basic Chinese (language used in the HealthCap); (v) have used any mobile app (not HT-related) in the previous 1 year; and (vii) aged between 18 and 80.

Patients will be excluded if any of the following criteria are met: (i) cannot provide informed consent; (ii) unwillingness to conduct HBPM or repeated ABPM; (iii) relative contraindications to ABPM (i.e. diagnosed atrial fibrillation, nighttime workers, occupational drivers, or patients with bleeding tendencies); (iv) have severe mental illnesses that impair their ability to use HealthCap, including those diagnosed with schizophrenia, dementia, or as being actively suicidal; (v) a diagnosis of other acute or chronic diseases that need regular physical assessments and/or medication changes (e.g. suboptimally controlled DM [e.g. glycosylated haemoglobin (HbA1c) ≥ 7%], depression requiring medications, active cancer); and (vi) predicted lifespan of <1 year.

All GOPCs within the New Territories East cluster and Hong Kong West cluster in Hong Kong are the eligible recruitment sites. Regarding the delivery of intervention, only the research team members who have been trained are eligible. All members in the research team have been trained on the study’s objectives and patient eligibility criteria, as well as to provide patients with instruction on how to use the HealthCap correctly. The physicians in charge have also been trained to utilise the HealthCap for monitoring patients’ blood pressure control and reviewing questionnaire responses before each patient’s visit. While patients are recruited by doctors, nurses, and the research team (including investigators and research assistants) during their visits to the clinics for hypertension care, eligibility will be further confirmed by the research assistants when patients are referred to the trial.

### Randomisation/blinding

Stratified randomisation with blocks of four or six will be used according to (i) the presence or absence of diseases that increased cardiovascular risk and required a lower BP target (<130/80 mmHg) (including DM, history of cardiovascular diseases, history of chronic kidney disease) and (ii) the number of anti-HT medications (<3 or ≥3) because the number of medications affects medication adherence and the likelihood of maintaining optimal BP control [17]. The randomisation sequence will be generated by an independent statistician, using the statistic programme Random Allocation Software, version 1.1.0, printed out, and sealed in light-opaque envelopes, which will only be opened by the research assistant after a participant’s eligibility is confirmed and the consent form is signed. Due to the nature of the intervention, blinding the investigators, participants, and healthcare providers will be infeasible after randomisation. However, the independent statisticians who will conduct the analysis will remain blinded.

### Intervention/control arm

#### Intervention arm: telemedicine

Patients will be: (i) given a validated HBPM device (ORMON HEM-7120) with appropriate cuff size, (ii) taught the HBPM technique, and (iii) taught to record HBPM readings using the HealthCap mobile app on their smartphones.

**Fig. 1:**
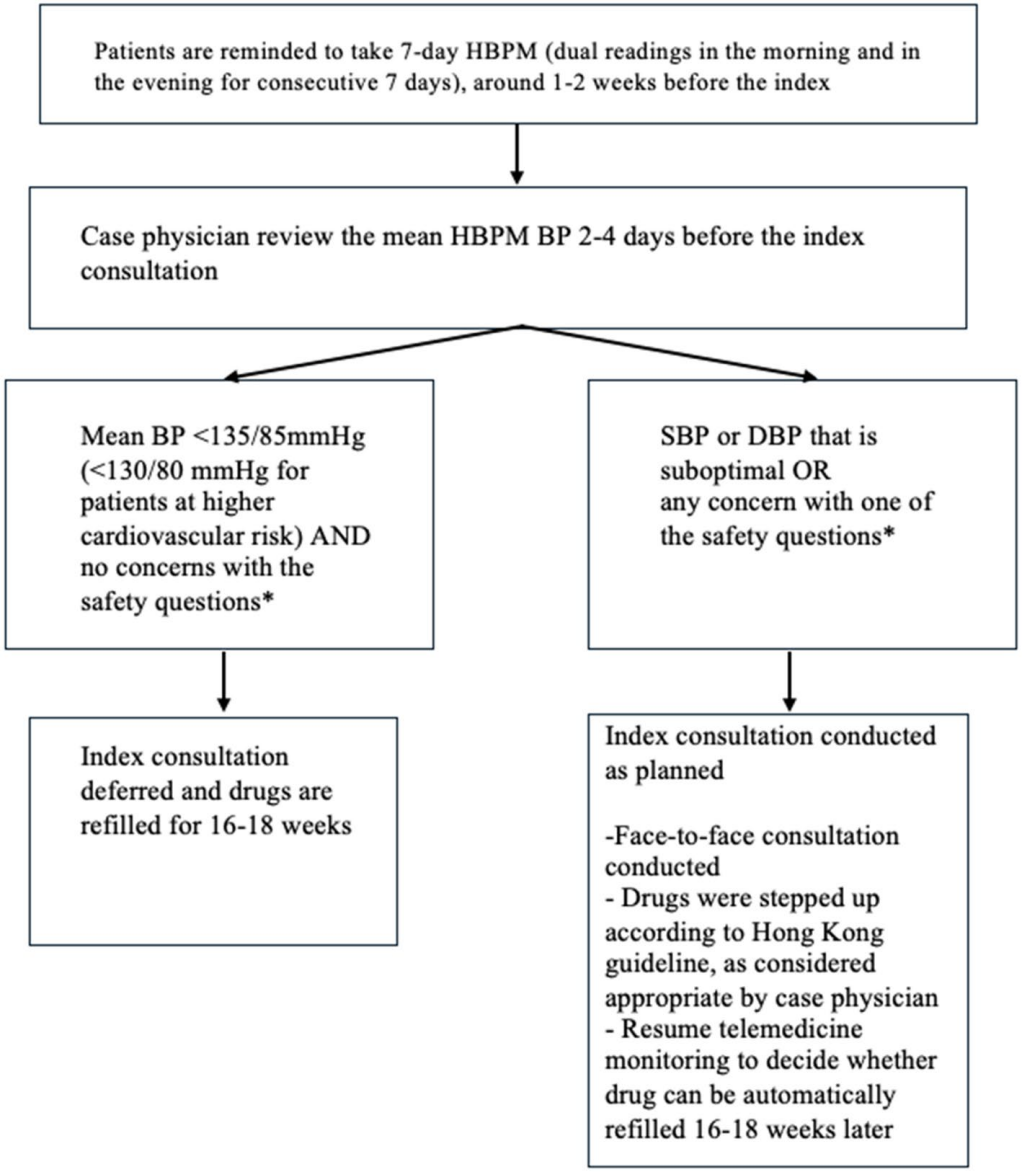
Flowchart for patients in the intervention (telemedicine) group. *Safety questions included the following: (i) self-reported good drug compliance and no drug side effect, (ii) absence of symptoms of complications (i.e. chest pain and hemiplegia), (iii) (only for patients with DM) absence of hyperglycaemia and hypoglycaemia symptoms, and (iv) no other health complaints

Participants randomised to intervention will be reminded to take dual BP readings in the morning and evening for 1–2 weeks before the index consultation. These BP readings will be automatically sent to a computer at the clinic. When the HBPM mean is optimal (i.e. <135/85 mmHg or <130/80 mmHg [for patients with cardiovascular diseases, renal diseases, and DM]), other parameters will be checked using an online questionnaire, and responses will be screened by the physicians, including (i) self-reported good drug compliance and no drug side effects, (ii) absence of symptoms of complications (i.e. chest pain and hemiplegia), (iii) absence of hyperglycaemia and hypoglycaemia symptoms (only for patients with DM), and (iv) no other health complaints that need consultation. When all the answers are negative, medications will be delivered to the patient by tracked mail, and the index physician appointment will be deferred for 16–18 weeks (the usual follow-up period for these patients is now 16–18 weeks). The index consultation will proceed as scheduled if BP is suboptimal or any of the safety questions screen is positive. HealthCap has an automatic feedback function. For example, if BP is dangerously high (i.e. SBP ≥ 180 or DBP ≥ 110 mmHg), HealthCap will recommend a recheck and advise attendance at the emergency department if BP remains elevated (Fig. 1).

HealthCap may also enhance self-management because it automatically reminds patients to measure BP regularly (every 1–2 weeks), provides feedback on patients’ BP values (compared to the threshold of elevated BP), and links to online healthcare educational resources.

#### Control arm

Participants will continue receiving routine care, including anti-HT drug prescriptions, from their regular physicians. In HK, patients with well-controlled HT are routinely seen every 16–18 weeks. Participants will also be given the same HBPM devices and taught the techniques. This is necessary because HBPM is a secondary outcome. According to the HK guidelines, all patients with HT are advised to regularly monitor their home BP, which can be considered as usual care [16]. However, the patients will not be taught any BP measurement algorithm (such as that used in the telemedicine group). They will also be asked not to download or use any new HT mobile apps during the study period. Additionally, the research assistant will ask if patients use any HT mobile apps at the baseline. The research assistant will then monitor and record the use of any new HT mobile apps at 6 months and 12 months.

In HK, all citizens have unlimited access to GOPCs and emergency departments for health problems. These services will not be limited for any participants in the current RCT. All participants are advised to seek medical help if BP becomes dangerously and persistently high (i.e. SBP ≥ 180 or DBP ≥ 110 mmHg) or in case of any suspected medical emergencies.

### Criteria for discontinuing or modifying allocation

Participants are encouraged to adhere to their assigned allocation as closely as possible. If any of the answers to the safety questionnaire are positive or the mean BP value from 7-day HBPM is suboptimal, the patient in the intervention group will receive usual doctor consultation. This consultation is part of the intervention and does not constitute a modification of allocation. Although there are no criteria for allocation modification, participants may withdraw from the study at any time if they choose to.

### Strategies to improve enrolment and adherence to interventions and follow-up

The recruitment rate will be regularly monitored. A trained research assistant will remind participants about adherence to interventions (e.g. 7-day home BP readings and filling in the safety questionnaire before index consultations in the HealthCap arm). Moreover, participants will be given supermarket coupons after they complete data collection at each timepoint (i.e. baseline, 6 months, and 12 months) to compensate the time and travelling cost, and to reinforce the patient adherence to the trial and data collection.

### Relevant concomitant care permitted or prohibited during the trial

Patients in the control group will be asked not to download or use any new HT mobile apps during the study period. There is no other specific concomitant care administered nor prohibited during the trial.

### Outcome measures

#### Primary outcome: daytime ABPM BP at 12 months

ABPM is the reference standard for BP measurements because of its superior reproducibility and predictability of cardiovascular outcomes [13, 14]. Mean daytime SBP will be the primary outcome of the RCT. SBP instead of DBP will be our primary outcome because SBP is more predictive of cardiovascular events than DBP, especially in the elderly [18]. WatchBP O3 (Microlife AG, Switzerland) has been validated by multiple HT societies (www.stridebp.org) and will be used in the current RCT. ABPM will be conducted in accordance with international guidelines (including the use of the non-dominant arm and appropriate cuff size) [19]. BP will be measured every 30 min for ≥24 h, and patients’ sleep diary will define the sleep duration. The readings will be considered valid if there are >70% valid readings overall, >20 valid awake, and >7 valid asleep BP readings in 24-h intervals [19]. Furthermore, erroneous and physiologically impossible BP readings are excluded (i.e. SBP outside the range of 50–240 mmHg and DBP outside the range of 40–140 mmHg) [19].

#### Secondary outcomes

Secondary outcomes will include variables related to other BP parameters, patient treatment adherence and self-efficacy, and health service utilisation. All secondary outcomes will be collected at baseline, 6 months, and 12 months unless specified otherwise. Acceptability will be further assessed by patient and physician interviews.

Blood pressure: This will include ABPM parameters (24-h/daytime/nighttime SBP and DBP) at 6 months and other ABPM parameters at 12 months (24-h/nighttime SBP/DBP and daytime DBP).

Furthermore, 7-day HBPM SBP/DBP will be measured using a validated device (ORMON HEM-7120; www.stridebp.org). Patients will be asked and reminded by the research assistant to measure dual readings in the morning and evening for 7 days in accordance with the local HT guidelines [16]. The mean SBP/DBP from the last 6 days will be used for analysis (BP readings from the first day can be higher due to anxiety, which is in accordance with local and international guidance) [12, 16]. This measurement algorithm was feasible and acceptable in our pilot RCT [9]. The minimal number of HBPM readings required for valid BP estimation is ≥12 BP readings [12]. HBPM may be more acceptable than ABPM in some patients whose sleep is disturbed by the ABPM or who have allergic reactions due to prolonged exposure to ABPM measurement cuffs [20]. Inclusion of HBPM as our secondary outcome will ensure successful out-of-office BP measurements in these patients.

Although less predictive of cardiovascular outcomes and less reproducible, office SBP/DBP will be collected at baseline and 12 months because office BP is still widely used in clinical practice. Various office BP devices are used in GOPCs and have been validated and maintained by the HK Hospital Authority. Measurement of office BP in GOPCs follows the HK primary care guidelines [16].

Health service utilisations and cost (collected at 12 months): Resource utilisation during the study period (including GOPC visits, emergency department visits, and hospitalisation) will be retrieved from the computerised clinical management system (CMS), and visits to private healthcare sectors, including private clinics and hospitals, will be self-reported. The number and types of anti-HT drugs will also be retrieved from CMS. Patients’ productivity loss (e.g. loss of work days due to doctors’ visits) will be self-reported. Their health-related quality of life will be assessed by the validated EQ-5D-5L at baseline, 6 months, and 12 months (needed for cost-effectiveness analysis if HealthCap is found superior to usual care; see under statistical method) [21].

Treatment adherence: This will be measured by the Treatment Adherence Questionnaire for patients with HT, which is validated in Chinese and contains measurements of adherence to medications, diet, stimulation, weight control, exercise, and stress reduction [22].

Self-efficacy: This will be measured using the validated 5-item self-efficacy scale specific to HT, which was found to have good internal validity (Cronbach’s alpha = 0.81) with a mean score of at least 9 (out of 10), signifying good self-efficacy [23].

Other physical parameters: Blood and urine tests will be collected at baseline and 12 months (serum creatinine, fasting glucose, HbA1c, and lipid levels [total cholesterol, total triglyceride, low-density lipoprotein, and high-density lipoprotein]), together with body mass index.

Satisfaction with HealthCap: All participants in the intervention group will be asked to rank their satisfaction with HealthCap and with the automatic drug refill process on a scale from 0 (completely dissatisfied) to 10 (completely satisfied) at 12 months. They will also be asked if they want to be recruited for an individual patient’s interview.

Acceptability: To assess the acceptability of the HealthCap system, around 30 patients with high (highest quartile score) and low (lowest quartile score) satisfaction will be invited for a patients’ interview until qualitative data saturation. Similarly, participating physicians (likely total number < 30) will be interviewed until qualitative data saturation if possible. Interviews will be conducted by the same trained research assistant face-to-face or by telephone. We will pilot test the patients’ interview guide on 2–3 patients prior to formal data collection.

Physicians will be asked about their experience of (i) introducing the HealthCap system to the patients, (ii) the drug auto-refill process and any relevant difficulties and concerns, (iii) a change in the doctor-patient relationship, (iv) any extra workload due to the use of the HealthCap system, (v) how HealthCap changes their usual practice and any perceived unmet patients’ health needs, and (vi) how likely that they will continue to promote and use the HealthCap system for these patients.

Similarly, patients randomised to use the HealthCap system will be asked about their experience with (i) a more frequent HBPM and self-management, (ii) any observed health changes and difficulties due to the HealthCap system, (iii) any unmet health needs during the last 1 year, (iv) the automatic drug refill process, (v) a change in the doctor-patient relationship, and (vi) how likely that they will continue to use the HealthCap system. The questions may be modified according to the quantitative results or any difficulty using HealthCap detected during the RCT. Data will be collected until saturation is reached.

Baseline data collection: Data on age, sex, marital status, educational level, smoking status (current smoker, ex-smoker, or never smoked), and alcohol use (assessed by the validated Alcohol Use Disorders Identification Test questionnaire) will be collected on recruitment [24]. Furthermore, eHealth literacy will be assessed using the validated Chinese 8-item eHealth literacy scale, which has high internal consistency (Cronbach’s alpha = 0.95) and has been used in Chinese with chronic diseases [25].

### Data collection and management

All research assistants who are involved in data collection will be trained in the correct use of instruments (e.g. ABPM) and questionnaires, as well as the retrieval of data. After each assessment, the files will be reviewed by research assistants to identify missing or erroneous data. Any missing information will be retrieved immediately from the instruments and the study participants. The data will be entered into the computerised database weekly or bi-weekly, depending on the recruitment rate. Data of participants at all timepoints will be collected even if they withdraw from the interventions, with their consent. The confidentiality of sensitive data (i.e. personal information) will be ensured by minimising the number of personnel who handle subject data. All data will be securely stored in password-locked computers and locked cabinets. For the questionnaire, data will be entered directly by patients online using research-grade software, Qualtrics. We will implement limits for responses to exclude impossible values, ensuring that the data is accurately captured in our database. All other data entries will undergo double data entry by two independent assessors to ensure accuracy and reliability. Each participant will be assigned a unique identification code for research data management. The code list can only be accessed by the research team and will be securely maintained by the principal investigator after the study is completed. Personal information will remain confidential throughout the study and will not be disclosed in future publications.

### Monitoring and adverse event management

There are no anticipated risks associated with the intervention. For participants using the HealthCap mobile app, index consultation will proceed as scheduled if BP is suboptimal or any of the safety questions screen is positive. HealthCap also has an automatic feedback function. For example, if BP is dangerously high (i.e. SBP ≥ 180 or DBP ≥ 110 mmHg), HealthCap will recommend a recheck and advise attendance at the emergency department if BP remains elevated.

In HK, all citizens have unlimited access to GOPCs and emergency departments for health problems. These services will not be limited for any participants in the current RCT. All participants are advised to seek medical help if BP becomes dangerously and persistently high (i.e. SBP ≥ 180 or DBP ≥ 110 mmHg) or in case of any suspected medical emergencies. In such circumstances, patients are asked to contact the study team at their earliest convenience. Any hospitalisations will be promptly reported to the relevant ethics committee within 24 h, or as soon as possible. Due to the low-risk nature of the study and that the funder did not allocate resources for a Data Monitoring Committee (DMC), this study will not have a DMC.

An interim report will be submitted by the principal investigator to the Health and Medical Research Fund, Hong Kong SAR, the funder of the current study only for its review and record (available at https://rfs1.healthbureau.gov.hk/english/funds/funds_hmrf/funds_hmrf_abt/funds_hmrf_abt.html). The report will include updates on the aims and objectives of the research, the timetable of work, and the achievements and major findings of the project to date. It will also detail expenditure and provide comments on the potential for disseminating research findings. Additionally, we will report on the recruitment rate, assess the balance between the two arms of the study, including any demographic imbalances that may be of concern, and report any interim results. Furthermore, we will document any trial-related adverse events and severe adverse events encountered during the study. The principal investigator will closely monitor participant safety during the study period. In the event of an adverse event, the principal investigator will review the case and report to the ethics committee for record and investigation. Although no harm to participants is expected from this study, we have consistently reported all severe adverse events to the ethics committee within 1 to 2 days of becoming aware of them. Should any concerns arise regarding the trial, the ethics committee has the authority to halt the trial at any time. Clinical trial insurance was purchased to cover compensation for participants who are harmed as a consequence of participation in this study. Financial auditing of the current study will be performed by the Chinese University of Hong Kong SAR.

### Sample size calculation

According to existing literature, a minimal clinically meaningful difference was set at an SBP difference of 2 mmHg [26]. By further setting type I error at 5% and type II error at 20%, and a standard deviation (SD) of 10 mmHg on ABPM (this is our primary outcome, and we observed this SD in our previous local studies) [27], the required sample size for this non-inferiority RCT will be 309. At the maximum dropout rate of 15%, 364 patients are required.

### Statistical analysis

The baseline characteristics of the participants in the two arms will be described using means with SD and proportions for continuous and categorical variables, respectively. The effect of the HealthCap telemedicine system on SBP measured by ABPM (our primary outcome) will be examined using analysis of covariance (ANCOVA) with baseline SBP measured by ABPM and the treatment group as the covariate, following the intention-to-treat (ITT) principle, that is, patients will be encouraged to conduct ABPM at 12 months regardless of whether they comply with the assigned intervention. A per-protocol (PP) analysis will be conducted for sensitivity analysis. PP analysis provides some protection for theoretical increases in the risk of type I errors (erroneously concluding non-inferiority). Our modified ITT (mITT) data comprised all patients according to and were included in the random allocation of complete data. We defined the PP population as participants who met the mITT definition but did not receive intervention treatments. Based on a previous meta-analysis, we set the non-inferiority margin for ABPM SBP at 2 mmHg [26]. We accepted the non-inferiority of telemedicine over usual care (in a 0.0125-level test after Bonferroni correction for multiple testing) if the lower bound of the two-sided 95% confidence interval (CI) (equivalent to the upper bound of the one-sided 97.5% CI) was within the non-inferiority margin. Subgroup analysis will be conducted in cases of unbalanced baseline characteristics and for patients with and without indications of a lower BP target (i.e. <130/80 mmHg). Additionally, missing data will be addressed through multiple imputation.

Qualitative data will be analysed by at least 2 investigators using inductive thematic analysis as described by Braun and Clarke [28]. Therefore, digital recordings will be transcribed verbatim. The recordings will be listened to several times, and the transcripts will be read and re-read by at least 2 investigators. After familiarisation, codes and then themes will be generalised. The investigator team will discuss the themes to determine and decide on any over-arching themes. The Consolidated criteria for Reporting Qualitative Research (COREQ) Checklist will be followed throughout the qualitative research part to ensure comprehensiveness and transparency in reporting qualitative findings [29].

Cost-effectiveness analysis will be performed from a societal perspective. We will collect direct medical costs (e.g. clinical visits) and direct non-medical costs (e.g. average HealthCap maintenance cost per person). Furthermore, self-reported indirect costs of productivity (mainly for absenteeism, using a human capital approach) and transportation to doctors’ visits will be recorded. Other cost items are listed in the section “Health service utilisations and cost”. Considering the targeted population (well-controlled hypertensive patients) and the nature of this study, we do not expect any changes in quality-adjusted life years (QALY). However, to confirm there is no intangible cost difference, we will use the 5-level EQ-5D (EQ-5D-5L) for QALY estimation. We will use a bootstrapping approach to assess the uncertainty of the incremental cost-effectiveness gain and to generate the cost-effectiveness acceptability curves to assess the estimated probability of cost-effectiveness in relation to possible values of the cost-effectiveness threshold.

## Discussion

This project has the potential to change clinical practice because patients with optimal BP can monitor their BP through a telemedicine system and will have less frequent clinical visits. This will empower primary care and allow effective and safe allocation of scarce medical resources to patients in need. It will also save patients time because of the long wait to see doctors and collect medications in GOPCs. Patients may also be empowered because they will play a proactive role in monitoring and managing their chronic illnesses. The HealthCap app also provides health educational material.

If the HealthCap system is found to be non-inferior to usual care: (i) further research could be conducted to implement HealthCap in routine clinical practice. The current project will also provide essential data (including patient and physician interviews) for implementation studies. (ii) As with other medical devices, studies should continuously monitor the long-term safety of patients during the broad implementation of HealthCap. Although the current study collected safety data (e.g. hospitalisation data), the study’s duration (i.e. 1 year) and sample size are modest to detect “hard” clinical outcomes (e.g. cardiovascular complications). (iii) The use of telemedicine can be extended to other stable chronic diseases (e.g. asthma/chronic depression). (iv) Future studies should also examine the role of the telemedicine system in Chinese patients with suboptimal BP control.

## Plan for communicating important protocol amendments

Any modification to the protocol, such as changes in sample size eligibility, study aim and objectives, intervention details, and methods, will be evaluated and approved by the Joint Chinese University of Hong Kong-New Territories East Cluster Clinical Research Ethics Committee and Institutional Review Board of the University of Hong Kong/Hospital Authority Hong Kong West Cluster before execution. In addition, the principal investigator will update the sponsor and funder, and the participating GOPCs regarding the approved amendment with the provision of a copy of the amended protocol.

## Dissemination of the study findings

In addition to publishing the protocol in an international journal for public access, the results of this RCT will be shared with the participants as well as disseminated through international peer-reviewed publications and conferences.

## Trial status

This trial is actively recruiting participants (114/365). This trial is ongoing and has a planned duration of 36 months. The recruitment began on 1 October 2024, and the estimated date of recruitment completion is 31 May 2026. The current protocol is version 2. If any changes needed to be made to the protocol, the relevant part of the study, as well as the record in the trial registry, will be updated (ClinicalTrials.gov, NCT06524180).

## Supplementary Information

Additional file 1. SPIRIT checklist.

Additional file 2. Detailed list of recruitment sites.

Additional file 3.

## Data Availability

The data will be available after the main manuscript is published upon reasonable request.

## Abbreviations

HT: Hypertension
HK: Hong Kong
BP: Blood pressure
RCTs: Randomised controlled trials
SBP: Systolic blood pressure
DBP: Diastolic blood pressure
HBPM: Home BP measurements
GOPC: General outpatient clinic
ABPM: Ambulatory blood pressure monitoring
SPIRIT: Standard Protocol Items: Recommendations for Interventional Trials
DM: Diabetes mellitus
CMS: Clinical management system
DMC: Data Monitoring Committee
SD: Standard deviation
ANCOVA: Analysis of covariance
ITT: Intention-to-treat
PP: Per-protocol
CI: Confidence interval
COREQ: Consolidated criteria for Reporting Qualitative Research
QALY: Quality-adjusted life years
EQ-5D-5L: 5-Level EQ-5D
