# Supplementary material for: Can TElemedicine system replace doctor consultations to Achieve non-inferior blood pressure in patients with Controlled Hypertension (TEACH)? Study protocol for a randomised controlled trial
Source: Trials. 2025 Dec 8;27:31. doi: 10.1186/s13063-025-09350-3 (PMC12797787; doi:10.1186/s13063-025-09350-3)
Supplement: Supplementary file 2 — Additional file 2. Detailed list of recruitment sites. [file 13063_2025_9350_MOESM2_ESM.docx]

**Additional document 2 detailed list of recruitment sites**

| Name of recruitment clinic | Address |
| --- | --- |
| Aberdeen Jockey Club Family Medicine Clinic | 10 Aberdeen Reservoir Road, Aberdeen |
| Ap Lei Chau Family Medicine Clinic | 161 Ap Lei Chau Main Street, Ap Lei Chau |
| North district Family Medicine Integrated Centre | North District Family Medicine Integrated Centre |
| Sai Ying Pun Jockery Club Family Medicine Clinic | 134 Queen's Road West, Sai Ying Pun |
| Lek Yuen Family Medicine Clinic | G/F, 9 Lek Yuen Street, Shatin |
