# Supplementary material for: Can TElemedicine system replace doctor consultations to Achieve non-inferior blood pressure in patients with Controlled Hypertension (TEACH)? Study protocol for a randomised controlled trial
Source: Trials. 2025 Dec 8;27:31. doi: 10.1186/s13063-025-09350-3 (PMC12797787; doi:10.1186/s13063-025-09350-3)
Supplement: Supplementary file 3 — Additional file 3. [file 13063_2025_9350_MOESM3_ESM.docx]

研究題目
遠距醫療系統能否取代醫生會診，讓控制良好的高血壓患者取得非劣效血壓控制？ 一項隨機對照試驗與成本最小化分析

TEACH informed consent form version 5 July 02, 2025

研究背景及重要性
香港中文大學賽馬會公共衛生學院設計及實施一項名為「遠距醫療系統能否取代醫生會診，讓控制良好的高血壓患者取得非劣效血壓控制？一項隨機對照試驗與成本最小化分析」的研究。這項研究旨為評估與常規醫生會診相比，遠距醫療系統（包含藥物補充）能否讓血壓控制良好的患者取得非劣效的血壓控制。如果該計畫發現遠距醫療系統（HealthCap）不遜色於常規護理，則可能會對醫療保健系统和政策、醫生的臨床實踐和患者產生積極影響。該項研究由醫療衞生研究基金資助。

研究對象及目的
本研究項目主要研究對象是血壓控制良好的高血壓患者，其目的是測試遠距醫療系統是否在血壓控制方面不遜色，以及確定該系統能否最大限度地降低成本並減少醫療保健使用率。

為避免對高風險患者產生額外風險，如參加者有以下狀況將會被排除：1）無法給予知情同意；2）不願意進行家庭血壓監測或重複動態血壓監測；3）動態血壓監測的禁忌症（即診斷為心房顫動，夜間工作人士，職業司機或有出血傾向的患者）；4）患有嚴重精神疾病，影響其使用遠距醫療系統的能力，包括被診斷出患有精神分裂症、認知障礙症或有自殺傾向的人；5）診斷出其他急性或慢性疾病，需要定期進行身體評估和/或改變藥物治療，如糖尿病控制不佳、糖化血紅蛋白(HbA1c)≥7%、需要藥物治療的抑鬱症、活性癌症；6）預期壽命<1年。

研究計劃內容
我們將招募364名患有高血壓患者參與一個隨機對照試驗，並將以1：1的比例隨機分配到遠距醫療系統或常規護理組。臨床結果將於計劃開始前、開始後6個月和12個月進行記錄並比較。

遠距醫療系統組
在研究期間，參加者將（1）獲得經過驗證的、具有適當袖帶尺寸的家庭血壓監測設備（ORMON HEM-7120），（2）學習如何使用家庭血壓監測，以及（3）學習如何使用智慧型手機上的遠距醫療移動應用程式（HealthCap）記錄家庭血壓監測讀數。

參與者在醫生會診前1-2週內早晚兩次測量和記錄血壓讀數。這些血壓讀數將自動發送到診所的電腦。對於沒有合併症的患者，診室外最佳日間血壓為<135/85 mmHg;對於患有增加心血管風險的合併症的患者，診室外日間最佳血壓為<130/85 mmHg。當家庭血壓監測平均值達到以上標準時，醫生將檢查參與者的線上問卷與回覆，包括（1）自我報告藥物依從性良好且無藥物副作;（2）無併發症症狀（即胸痛和偏癱）;（3）無高血糖和低血糖症狀（僅適用於糖尿病患者）;以及（4）沒有其他需要諮詢的健康投訴。當所有答案均為否定時，藥物將透過追蹤郵件交付給患者，並且醫生會診將推遲16-18星期。如果血壓控制不理想或有任何安全問題回答為肯定，醫生會診則將按計劃進行。

參加者將在計劃開始前、開始後6個月和12個月進行動態血壓監測和填寫問卷（包括病人治療依從性、自我效能以及與健康相關的生活品質等方面）。參加者將在計劃開始前和開始後12個月進行驗血、尿檢、量度體重指標。參加者還將在計劃開始後12個月填寫關於衛生服務使用和對HealthCap及自動分藥物補充過程滿意程度的問卷。部分參與者還將在計劃開始後12個月接受關於使用HealthCap感受的訪問。

常規護理組
在研究期間，參加者將（1）獲得經過驗證的、具有適當袖帶尺寸的家庭血壓監測設備（ORMON HEM-7120），（2）學習如何使用家庭血壓監測。參與者將繼續接受臨床醫生的常規護理，包括降血壓藥處方。在香港，高血壓控制良好的患者通常每16-18周就診一次。參與者還將獲得相同的家庭血壓監測設備並教授相關技術。參與者將被要求在研究期間不要下載或使用任何關於高血壓的流動應用程式。

參加者將在計劃開始前、開始後6個月和12個月進行動態血壓監測和填寫問卷（包括病人治療依從性、自我效能以及與健康相關的生活品質等方面）。參加者將在計劃開始前和開始後12個月進行驗血、尿檢、量度體重指標。參加者還將在計劃開始後12個月填寫關於衛生服務使用的問卷。

參與研究的影響
本計劃只涉及遠距醫療系統，目前未有任何資料顯示此方法帶來的任何不良反應。

自願參與/中途退出
是次研究的參與屬自願性質，閣下有權選擇是否參與。即使您在此提供了電子簽名，閣下亦有權拒絕回答問卷中任何問題，或隨時退出是次研究，而絕對不會影響閣下現享有的醫療服務及法律權益。請詳細閱讀下一頁，如有任何疑問，請聯絡負責同事邢先生(Tel. 57414114)

費用
研究人員不會向閣下索取任何費用。於開始前、開始後6個月及12個月完成評估的參與者，每次可獲贈100元超市禮券。

個人資料收集
閣下所提供的個人資料會絶對保密，並只會用作整體統計分析及報告之用，個別人士的身份絕不會被披露。本調查中收集的所有個人數據（例如姓名，性別，年齡等）將在研究後保存3年。我們之後會對臨床數據進行數據屏蔽（刪除所收集的臨床數據的個人信息部分）。屏蔽後的臨床數據將被永久保留用於將來數據分析目的。有需要的話，每位研究參與者都有權利獲得其個人的數據以及公開報告的研究結果。我們將在數據庫中存儲和封鎖所有收集的個人信息。只有特定幾個研究者才有數據庫的訪問權。我們將有一個嚴格的數據庫登錄程序，同時將定期更改密碼，以防止數據洩漏。

根據香港法律（特別是「個人資料（私隱）條例」，第486章），閣下有權對您個人資料進行保密，如在本項研究中或與本項研究有關的個人資料的收集、保管、保留、管理、控制、使用（分析或比較）、在香港內外轉讓、不披露、消除和/或任何方式處理。請注意，香港中文大學－新界東醫院聯網臨床研究倫理聯席委員會和香港大學及醫院管理局港島西醫院聯網研究倫理委員會是獲授權的機構，可以訪問與本研究相關的受試者記錄，以進行倫理審查。如有任何問題，閣下可以諮詢隱私資料私隱專員或致電到其辦公室（電話號碼：2827-2827），以適當監管或監督閣下個人資料保護，以便閣下能完全認識和瞭解確保遵守法律保護隱私資料的意義。

本研究負責人為李錦培教授(Prof. Eric Lee電郵：lkp032@cuhk.edu.hk)，如有任何查詢，歡迎致電:2252-8463。

同意參與該項研究，閣下明確作出以下授權：
• 為了監督該項研究，授權主要研究者及其研究團隊和倫理委員根據本項研究和此授權確認的內容規定的方式獲得、使用並保留您的個人資料；及
• 為了檢查和核實研究資料的完整性、評估研究協定與相關要求的一致性，授權相關的政府機構（如香港衛生署）可獲得您個人資料。

感謝您考慮參與這項研究和問卷調查。如您是在新界東醫院聯網參與這項研究，您可以聯絡香港中文大學－新界東醫院聯網 臨床研究倫理聯席委員會，電話：3505-3935，查詢任何有關研究倫理的問題。如您正在香港西醫院聯網參與這項研究，您可以聯絡香港大學及醫院管理局港島西醫院聯網研究倫理委員會，電話：2255-4086，查詢任何有關研究倫理的問題。

本人已經閱讀有關此研究計劃之詳情，清楚了解計劃之一切程序，亦明白本人的約定及承擔。

本人明白是次研究計劃的目的、方法及資料運用

本人明白所有收集得來的資料將由研究員保管，絕對保密，並且只作研究之用

本人明白所有參加者的身份不會被公開

本人明白本人有權隨時退出此研究計劃，而絕對不會影響本人現享有的醫療服務及法律權益。

日期

本人在此聲明，本人自願參與「遠距醫療系統能否取代醫生會診，讓控制良好的高血壓患者取得非劣效血壓控制？一項隨機對照試驗與成本最小化分析」研究

您的中文姓名

您的英文姓名

您的聯絡電話 (可以WhatsApp溝通)

Email (如有)
